# Metastable Resting State Brain Dynamics

**DOI:** 10.3389/fncom.2019.00062

**Published:** 2019-09-06

**Authors:** Peter beim Graben, Antonio Jimenez-Marin, Ibai Diez, Jesus M. Cortes, Mathieu Desroches, Serafim Rodrigues

**Affiliations:** ^1^Communication Engineering, Institute of Electrical Engineering and Information Science, Brandenburg University of Technology Cottbus – Senftenberg, Cottbus, Germany; ^2^Computational Neuroimaging Lab, BioCruces-Bizkaia Health Research Institute, Barakaldo, Spain; ^3^Department of Radiology, Gordon Center for Medical Imaging, Harvard Medical School, Massachusetts General Hospital, Boston, MA, United States; ^4^Neurology Department, Harvard Medical School, Boston, MA, United States; ^5^Neurotechnology Laboratory, Tecnalia Health Department, Derio, Spain; ^6^Ikerbasque - the Basque Foundation for Science, Bilbao, Spain; ^7^Department of Cell Biology and Histology, University of the Basque Country, Leioa, Spain; ^8^MathNeuro Team, Inria Sophia Antipolis Méditerranée, Valbonne, France; ^9^Université Côte d'Azur, Nice, France; ^10^Mathematical, Computational and Experimental Neuroscience, Basque Center for Applied Mathematics, Bilbao, Spain

**Keywords:** resting state, recurrence structure analysis, metastability, BOLD fMRI, diffusion tensor imaging, brain hierarchical atlas

## Abstract

Metastability refers to the fact that the state of a dynamical system spends a large amount of time in a restricted region of its available phase space before a transition takes place, bringing the system into another state from where it might recur into the previous one. beim Graben and Hutt (2013) suggested to use the recurrence plot (RP) technique introduced by Eckmann et al. (1987) for the segmentation of system's trajectories into metastable states using recurrence grammars. Here, we apply this recurrence structure analysis (RSA) for the first time to resting-state brain dynamics obtained from functional magnetic resonance imaging (fMRI). Brain regions are defined according to the brain hierarchical atlas (BHA) developed by Diez et al. (2015), and as a consequence, regions present high-connectivity in both structure (obtained from diffusion tensor imaging) and function (from the blood-level dependent-oxygenation—BOLD—signal). Remarkably, regions observed by Diez et al. were completely time-invariant. Here, in order to compare this static picture with the metastable systems dynamics obtained from the RSA segmentation, we determine the number of metastable states as a measure of complexity for all subjects and for region numbers varying from 3 to 100. We find RSA convergence toward an optimal segmentation of 40 metastable states for normalized BOLD signals, averaged over BHA modules. Next, we build a bistable dynamics at population level by pooling 30 subjects after Hausdorff clustering. In link with this finding, we reflect on the different modeling frameworks that can allow for such scenarios: heteroclinic dynamics, dynamics with riddled basins of attraction, multiple-timescale dynamics. Finally, we characterize the metastable states both functionally and structurally, using templates for resting state networks (RSNs) and the automated anatomical labeling (AAL) atlas, respectively.

## 1. Introduction

Mapping the brain's functional-structural relationship remains one of the most complex challenges in modern neuroscience (Park and Friston, 2013), in part due to the highly dynamic multi-scale nature of the brain's processes and structures as observed by different measurement modalities, which leads to technical and mathematical difficulties for establishing dynamically invariant relations across scales. As a result, which precise function emerges at the macro-scale (as measured by BOLD signals) from the underlying static neuronal architecture is not yet fully understood. In fact, this rests on a many-to-one function-structure relationship, which is hard to resolve and therefore novel methodologies are demanded. The present study addresses aspects of this macro-scale question by leveraging on recent developments of novel data-driven computational methods, which weeds out recurrent dynamical states from times series and associates to optimal brain structures, thus resolving the function-structure of the so-called resting state networks (RSNs) (Raichle et al., 2001; Fox et al., 2005; Diez et al., 2015; Smitha et al., 2017).

Several methodological advances are making strides in unveiling the macro-scale organization of the brain, comprising both hierarchical brain network structures and functions. These methods classify brain's macro-scale organization under the following terms: *structural connectivity* (resolved from diffusion tensor imaging techniques—DTI), *functional connectivity* (determined by statistical dependencies from BOLD signals) and *effective connectivity* (deduced by causality measures from BOLD signals). The neural structures and patterns of dynamical similarity are represented by e.g., the connectivity matrices. Specifically, for *structural connectivity, functional connectivity*, and *effective connectivity* the entries of the network's connectivity matrix indicate the anatomical links (white-matter tracts connecting different gray matter regions), the correlation strength and the causal strength between pairs of imaging regions of interest, respectively (Alonso Montes et al., 2015). Subsequently, features of this matrix can be exploited by using for example standard tools from linear algebra that rely upon spectral analysis (e.g., invariants such as eigenvalues) and other related/complementary methods, such as independent component analysis (ICA) (Bell and Sejnowski, 1997), partial least squares (PLS) (Krishnan et al., 2011), and many more. These procedures extract meaningful and independent quantities and thus decompose features within the connectivity matrix. However, mapping between these different matrices and additionally accounting for temporal dynamics is of paramount interest and an active research area in brain mapping.

To reduce the complexity of this issue, neuroscientists have primarily focused on a precise context provided by the RSNs. This global brain dynamics (measured from the observable fMRI) emerges while a subject is at rest and can be decomposed as a superposition of multiple activation patterns (Bell and Sejnowski, 1995; Beckmann and Smith, 2005; Vergun et al., 2016). Despite the simplicity of the context in which these brain activity patterns are generated, RSNs dynamics is rich and complex. Indeed, different RSNs have been associated to specific cognitive networks, for example, visual networks, sensory-motor networks, auditory networks, memory (default mode) networks, executive control networks, and some others (see for instance Beckmann et al., 2005 and references therein). This has led to the hypothesis that underlying such activation patterns (often recurring) is the existence of stable switching attractors that enhance information maintenance and facilitates cognitive transitions (Vidaurre et al., 2017; Iraji et al., 2018; Shine et al., 2018). Moreover, it is observed that the base resting state network is well conserved across subjects (Damoiseaux et al., 2006).

The mapping between structural connectivity and functional connectivity of RSNs (without considering temporal dynamics) was recently investigated by some authors of the present study and, as a by-product, a novel brain hierarchical atlas was identified (Diez et al., 2015). This was achieved by starting from the hypothesis that segregated functions are associated with distinct brain regions and both structure and functions have a hierarchical modular organization. This provided a tree (dendrogram) structure where the leaves of the tree (first-order nodes) associated to voxel measurements represent self-correlation (i.e., singleton) for the functional connectivity matrix and self-connectivity for the structural connectivity matrix. Moving up the tree corresponds to pairing up tree leaves that have strong correlations or connectivity (for functional connectivity and structural connectivity, respectively), thus forming higher-order nodes (or modules). Recursively, these modules are paired until the entire brain is represented by a mother node. It was shown that for about twenty modules maximal similarity between structural modules and functional ones was achieved and precisely these modules define the novel *brain hierarchical atlas* (BHA), which also explains the structure-function mapping in RSNs. However, the dynamical evolution of RSNs over time was not investigated and in fact has never been exploited directly from data, though indirect approaches have been explored. For example, computational brain network models have been proposed in attempt to reveal fundamental principles of RSNs (Hansen et al., 2015; Deco et al., 2017; Surampudi et al., 2019), and some of these models predict (for instance) that the human brain during resting state operates at maximum metastability, i.e., in a state of maximal network switching (also observed in EEG Roberts et al., 2019). Moreover, it is conjectured that information flow in the brain is guided by ordered sequences of metastable states (Friston, 1997; Rabinovich et al., 2008; Kelso, 2012; Tognoli and Kelso, 2014; Fingelkurts and Fingelkurts, 2017).

The present work takes a step further and considers a direct approach by employing a data-driven computational method to investigate the stable switching attractors hypotheses of RSNs, leveraging a recently developed method, called *recurrence structure analysis* (RSA) (beim Graben and Hutt, 2013, 2015; beim Graben et al., 2016). We note however that the proposed framework is not the only method for extracting reproducible time-resolved networks from data as very recently a competing framework based on *dynamic mode decomposition* was proposed (Kunert-Graf et al., 2018). Briefly, our method utilizes advanced theories of dynamical systems and time series analysis, attempting to extract optimal symbolic dynamics from time series observations that display transient and recurrent states (i.e., metastable states). This is achieved by building upon Poincaré's recurrence theorem, on the one hand, which states that trajectories of a complex dynamical system visit frequently certain regions of their available state space in the course of time and by the so-called *recurrence plot method* (RP), on the other hand, allowing visualization and matrix identification of recurrent states. These are then transformed to symbolic space by introducing *recurrence grammars*, which map state space trajectories onto symbolic sequences (beim Graben and Hutt, 2013, 2015; beim Graben et al., 2016). This is carried out by constructing state space partitions that are *maximally metastable*, based on the assumption that the discretized symbolic dynamics should be approximately Markovian. Thus combining the structure-function modules of the novel brain hierarchical atlas with optimized recurrence structure analysis, opens for the first time new avenues to identifying resting state networks with time-dependent recurrent cognitive states.

## 2. Methods

In this section we review our methods for the data acquisition, preprocessing, structure-function clustering and recurrence structure analysis.

### 2.1. Data Acquisition

Data from *N* = 30 healthy subjects (14 males) with age between 22 and 35 were used in this study. Data were provided by the Human Connectome Project, WU-Minn Consortium (Principal Investigators: David Van Essen and Kamil Ugurbil; 1U54MH091657) funded by the 16 NIH Institutes and Centers that support the NIH Blueprint for Neuroscience Research; and by the McDonnell Center for Systems Neuroscience at Washington University. The acquisitions were conducted on the Connectome Skyra, which is a customized Skyra platform with 100mT/m gradients for diffusion encoding and 42mT/m gradients for imaging. High resolution T1 anatomical images and functional images were used in this study.

High-resolution anatomical MRI was acquired using a T1-weighted 3D MPRAGE sequence with the following parameters: TR= 2,400 ms; TE = 2.14ms; TI = 1, 000ms; Flip angle = 8°; FOV = 224 × 224; Voxel size = isotropic 0.7mm; BW = 210Hz/Px; iPAT: 2; Acquisition time 7 min and 40 s.

To measure changes in blood-oxygenation-level-dependent (BOLD) T2^*^ signals a gradient-echo EPI sequence was used to acquire 1,200 volumes (approximately 15 min). The acquisitions were performed with the following parameters: TR = 720ms, TE = 33.1ms; Flip Angle 52; field of view 208 × 180mm (RO × PE); 104 90 (RO × PE) matrix; 72 slices with 2.0mm isotropic voxels; multiband factor 8; echo spacing 0.58ms; and BW 2, 290Hz/Px.

For further information on acquisition parameters and scanning paradigms see the Human connectome documentation^1^.

### 2.2. Data Preprocessing

Functional data were preprocessed using FSL (FMRIB Software Library, version 5.0) and AFNI, following a procedure similar to previous work (Diez et al., 2017; Rasero et al., 2017, 2019; Stramaglia et al., 2017; Bonifazi et al., 2018; Camino-Pontes et al., 2018). First, slice-time correction was applied to the fMRI. Next, each volume was aligned to the middle volume to correct for head movement artifacts. After intensity normalization, we removed the effect of confounding factors: movement time courses, the average cerebrospinal fluid (CSF) signal and the average white matter signal, followed by a bandpass filter between 0.01 and 0.08Hz. The functional data were normalized to the MNI152 brain template, with a voxel size of 3 × 3 × 3mm^3^ and spatially smoothed with a 6mm full-width-at-half-maximum (FWHM) isotropic Gaussian kernel. In addition to head motion correction, we performed scrubbing, which means that all time points with framewise displacement greater than 0.5 were interpolated by a cubic spline (Yan et al., 2013).

### 2.3. Brain Hierarchical Atlas

**Figure 1:**
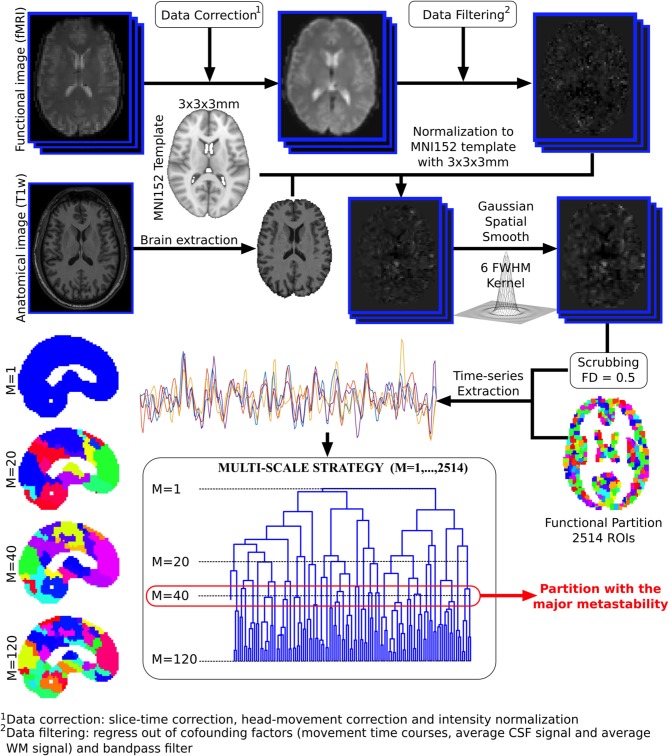
Functional image preprocessing pipeline. Dual acquisition is needed, high-resolution anatomical images (T1) and functional images at rest. Following state-of-the-art pipeline of neuroimaging preprocessing, time series of the blood oxygenation level dependent (BOLD) signal were obtained for each region of interest (ROI), defined by a functional atlas of 2,514 ROIs. Finally, the time series were averaged using different partitions of the brain hierarchical atlas (BHA). The partition with maximal metastability was the one with *M* = 40 modules (see section 2.4 for details).

Voxel time series were grouped using the brain hierarchical atlas (BHA), recently developed by Diez et al. (2015)^2^. The use of the BHA guarantees three conditions simultaneously: (1) That the dynamics of voxels belonging to the same module is very similar, (2) that those voxels belonging to the same module are structurally wired by white-matter streamlines; and (3) when varying the level of the hierarchical tree, it provides a multi-scale brain partition, where the highest dendrogram level *M* = 1 corresponds to all 2,514 regions belonging to a single module (coincident with the entire brain), whereas the lowest level *M* = 2,514 corresponds to 2,514 isolated modules (all of them composed of only one region). Figure 1 illustrates the functional image preprocessing pipeline.

### 2.4. Recurrence Structure Analysis

The recurrence structure analysis (RSA) exploits the *Poincaré recurrence theorem*, the *recurrence plot* (RP) technique introduced by Eckmann et al. (1987) and Markov state modeling to extract symbolic dynamics and recurrent metastable states from time series data^3^.

Consider a discretely sampled trajectory $X=\{x_{t}\in {\mathbb{R}}^{M}\;|\;0\leq t\leq T\}$ of duration *T* where ℝ^*M*^ denotes the *M*-dimensional phase space of the system. In our case, the states $x_{i}$ are obtained as normalized, voxel-averaged resting state fMRI (rsfMRI BOLD) measurements such that *M* is the number of structure-function modules (SFM) from the BHA analysis of section 2.3 (Diez et al., 2015).

The RP is a binary time-by-time matrix ***R*** indicating recurrence events, *R*_*ik*_ = 1, when two states $x_{i},x_{j}\in {\mathbb{R}}^{M}$ at times *i* > *j* are detected as being recurrent, i.e., state ***x*_*j*_** falls into a ball of radius ε > 0 centered around state ***x*_*i*_**:

(1) $$R_{ij}=\{\begin{array}{ll} 1 & \text{ if }x_{j}\in B_{\varepsilon }(x_{i})\\ 0 & \text{ else } \end{array}$$

with $B_{\varepsilon }(x_{i})=\{x\in {\mathbb{R}}^{M}\;|\;d(x,x_{i})<\varepsilon \}$ and an appropriately chosen metric or distance function *d*(***x,y***). Here, we use the cosine distance

(2) $$\begin{array}{r} d_{\cos}\left(x,y\right)=1-x\cdot y \end{array}$$

for normalized states, ∥x∥=∥y∥=1.

According to beim Graben and Hutt (2013, 2015), the RP (1) can be interpreted as a rewriting grammar, called the *recurrence grammar*, acting on sequences of time indices *s*_*i*_ = *i* in the following sense. If the system is recurrent at time points *i, j* (*R*_*ij*_ = 1) and if *i* > *j*, then a grammar rule *i* → *j* is defined, replacing the larger time index *i* in the sequence *s* by its smaller recurrent counterpart *j*. Moreover, if the system is recurrent at time points *i, j, k* (*R*_*ij*_ = 1, *R*_*ik*_ = 1) and if *i* > *j* > *k*, two grammar rules *i* → *k* and *j* → *k* are introduced that replace the two larger indices *i, j* in the sequence *s* by the smallest one *k*. Applying this grammar at least twice to the sequence *s* yields a transformed sequence *s*′ whose indices indicate the distinguished metastable system states.

A metastable state *S*_*k*_ is then rendered as a cloud of all states from the trajectory *X* that have the same index *k* in the sequence *s*′, that is,

(3) $$\begin{array}{r} S_{k}=\{x_{i}\in {\mathbb{R}}^{M}\;|s_{i}^{\prime }=k\}. \end{array}$$

The metastable states partition the phase space of the system into mutually disjoint equivalence classes. However, the discretization of the phase space and segmentation of system's trajectories into metastable states depends on a free parameter, the ball size ε. Determining the optimal ε value is pivotal as it enables to explain the time series observations. Several methods have been proposed to optimize ε (beim Graben and Hutt, 2013, 2015) but a more robust approach is based on Markovian optimization (beim Graben et al., 2016). Specifically, it assumes that the time series can be described by a Markov state model expressed via a transition matrix, $P=\left(p_{ij}\right)$, which specifies conditional transition probabilities from metastable state *S*_*j*_ into state *S*_*i*_,

(4) $$p_{ij}(\varepsilon )=\mathrm{Pr}(x_{t+1}\in S_{i}\;|\;x_{t}\in S_{j})\;,$$

where $x_{t}$ is the state at time *t* and $x_{t+1}$ its immediate successor in the given sampling. Moreover, it is assumed that the system spends most time in its respective metastable states and that the transitions from one metastable state into a transient regime and back into (another) metastable state are uniformly distributed (according to a *maximum entropy principle*). These combined assumptions enable the derivation of the following utility function

(5) $$u(\varepsilon )=\frac{1}{n+2}\left[\text{tr }P(\varepsilon )+h_{r}(\varepsilon )+h_{c}(\varepsilon )\right]$$

where tr ***P***(ε) is the trace of the transition matrix and

(6) $$\begin{array}{r} \begin{array}{cc} h_{r} & =-\frac{1}{\log\left(n-1\right)}\overset{n-1}{\underset{j=1}{\sum }}p_{0j}^{\prime }\log p_{0j}^{\prime },\\ h_{c} & =-\frac{1}{\log\left(n-1\right)}\overset{n-1}{\underset{i=1}{\sum }}p_{i0}^{\prime }\log p_{i0}^{\prime }. \end{array} \end{array}$$

are entropies (for the row and column of the transition matrix) with renormalized transition probabilities

(7) $$\begin{array}{r} \begin{array}{cc} p_{0j}^{\prime } & =\frac{p_{0j}}{\sum_{j=1}^{n-1}p_{0j}},\\ p_{i0}^{\prime } & =\frac{p_{i0}}{\sum_{i=1}^{n-1}p_{i0}}. \end{array} \end{array}$$

An optimal partition is then obtained through

(8) $$\begin{array}{r} \varepsilon^{*}=\arg\underset{\varepsilon }{\max}u\left(\varepsilon \right), \end{array}$$

entailing a *maximally metastable* Markov state model; for more details see beim Graben et al. (2016). The number of segments *n*(ε^*^) characterizes the “complexity” of the transition model comprising one distinguished transient and hence *n* − 1 metastable states (beim Graben et al., 2016).

Next, we have to consider ensemble analysis. Instead of looking at a single trajectory *X* we are concerned with an ensemble of *N* trajectories *E* = {*X*_*m*_ | 1 ≤ *m* ≤ *N*}, which in our case refers to rsfMRI time series from *N* = 30 individual subjects. As our data are normalized to a unit hypersphere, we are able to compare metastable segments from different subjects. This is achieved by collecting all metastable states $S_{k}^{\left(m\right)}$ from all individuals *m* and calculating their pairwise Hausdorff distances

(9) $$\begin{array}{r} D_{ij}=\max\{\max\{\delta (y,S_{j})\;|\;y\in S_{i}\},\max\{\delta (y,S_{i})\;|\;y\in S_{j}\}\} \end{array}$$

according to Hutt and beim Graben (2017). Here,

(10) $$\begin{array}{r} \delta (x,A)=\min\{d(x,y)\;|\;y\in A\} \end{array}$$

measures the “distance” of the point ***x*** from the compact set *A* ⊂ *X*. Note that the Hausdorff distance of two overlapping compact sets vanishes. Again, we use the cosine distance (2) for our normalized data here. Thresholding the distance matrix ***D*** with respect to a parameter θ > 0, yields another binary matrix ***Q*** as follows

(11) $$Q_{ij}=\{\begin{array}{ll} 1 & \text{ if }D_{ij}<\theta \\ 0 & \text{ else } \end{array}$$

which induces another recurrence grammar from which a Hausdorff clustering of metastable states is obtained; for further details see Hutt and beim Graben (2017). The threshold θ must be chosen in such a way that the entire ensemble *E* is covered by a minimal set of metastable states.

Finally, we have to express the metastable states either in terms of resting state networks (RSN) or in terms of anatomical structures (AAL). To that aim, Diez et al. (2015) have presented overlap matrices M∈M(M×K) relating *M* structure-function modules to *K* nodes in the RSN or AAL representations, respectively. Here we consider the matrix ***M*** as a mixing matrix mapping a module state vector $x\in {\mathbb{R}}^{M}$ onto a network state $y\in {\mathbb{R}}^{K}$ through

(12) $$\begin{array}{r} y=M\cdot x. \end{array}$$

For each metastable state *S*_*i*_, which is actually given as a spherical distribution of sampling points, we select a representative point. Hutt and beim Graben (2017) suggested to use the ensemble mean, i.e., the barycenter of the distribution. Here, however, we choose the vector

(13) $$x_{i}^{*}=\arg\underset{x\in S_{i}}{\max}\|x{\|}_{\infty }$$

maximizing the maximum norm of the distribution for enhancing the contrast between metastable states. We project these particular representatives $x_{i}^{*}$ onto the RSN and AAL representations, according to

(14) $$\begin{array}{r} y_{i}^{*}=M\cdot x_{i}^{*}, \end{array}$$

with ***M*** either the RSN or AAL mixing matrix, respectively.

## 3. Results

**Figure 2:**
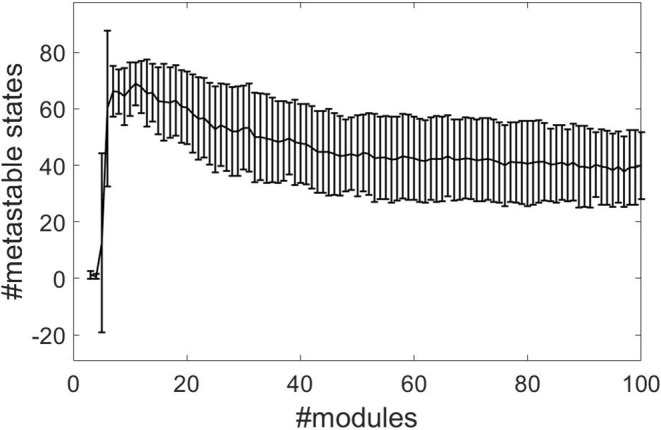
Segmentation complexities measured as number of metastable states against number of structure-function modules for normalized data using the cosine distance.

We combine the time-independent structure-function and HBA analysis of Diez et al. (2015) with the time-dependent recurrence structure analysis. We compute the optimal number of metastable states *n* − 1 determined by the RSA as a measure of complexity (beim Graben et al., 2016) for all subjects and for module numbers ranging from 3 to 100 from normalized rsfMRI BOLD signals. Figure 2 shows the results, where the error bars indicate one standard deviation of the complexity averaged over all subjects. The RSA stabilizes around *M* = 40 metastable states.

**Figure 3:**
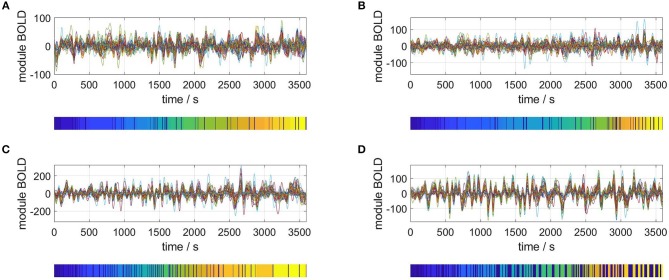
Selection of segmented rsfMRI time series. Upper panels: rsfMRI time series averaged over 40 modules, lower panels: symbolic sequences *s*′ resulting from optimal partitions into metastable states. Selected subjects: **(A)** #1, **(B)** #7, **(C)** #18, **(D)** #30.

Carrying out the RSA of an *M* = 40 dimensional phase space for four representative subjects gives the results plotted in Figure 3. For each plot, the upper panel shows the *M* time series of the rsfMRI BOLD signal before normalization. The lower panels depict the resulting segmentation into metastable states, i.e., the symbolic sequences *s*′.

**Figure 4:**
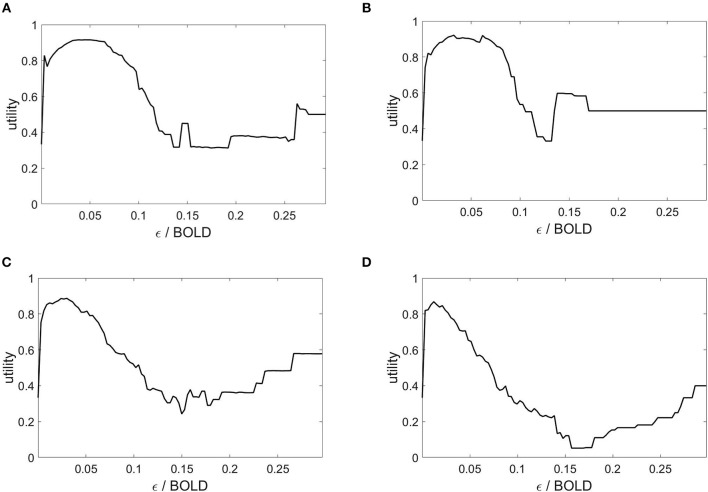
Markov utility functions from averaged rsfMRI time series over 40 modules. Subjects: **(A)** #1, **(B)** #7, **(C)** #18, **(D)** #30.

The state space partitioning and symbolic segmentation is achieved by optimizing the Markov criterion (8). The corresponding utility functions are plotted in Figure 4 and we observe that for each plot there exists a global maximum, establishing an optimal ball size for the symbolic segmentation around ε = 0.05.

**Figure 5:**
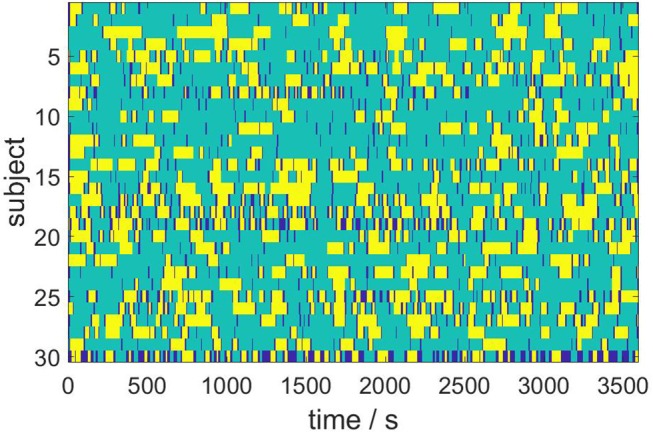
Bistable population resting state dynamics as resulting from Hausdorff clustering.

We next consider the ensemble dynamics from the population of *N* = 30 subjects by employing the Hausdorff clustering (9) and optimally thresholding the distance matrix (11) to obtain a minimal set of metastable states that covers the entire ensemble. For the choice θ = 0.8, we get a bistable segmentation of the resting state dynamics, specifically, into two metastable states and one transient state, which is depicted in Figure 5. Here, yellow and turquoise indicate the two metastable states, while dark blue represents transients.

**Figure 6:**
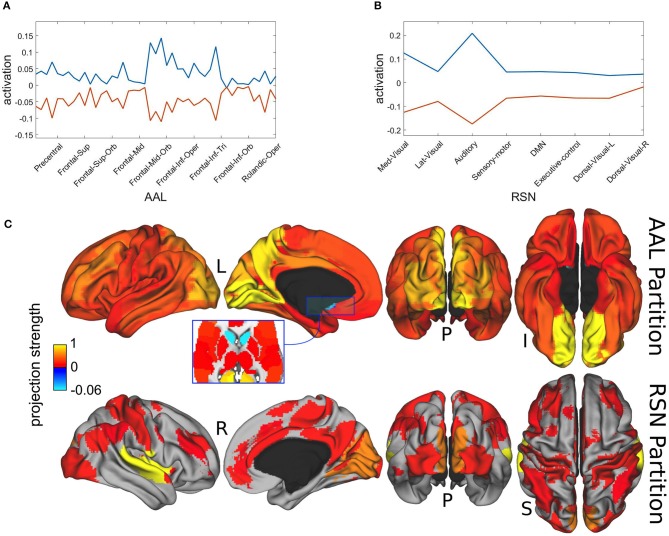
Spatial projections of metastable brain resting states. **(A)** Projection onto AAL regions. Notice that just for illustration purposes, although the spatial projection has been performed over all the 45 AAL brain regions, some of the labels have been removed from the x-axis. Brain areas corresponding to maximum projections (written in text) were lingual, calcarine, precuneus, occipital and cingulate cortices. **(B)** Projection onto RSN regions. Maximum projections occurred for the auditory and medial visual networks. **(C)** Brain localization of blue attractor with regard to AAL and RSN partitions. Notice that projection strength might have positive or negative values depending on the direction of the basis vectors.

In Figure 6 we present the spatial distributions of both metastable population states, resulting from the projection of the most distinctive representatives $x^{*}$ onto the AAL atlas in Figure 6A and with respect to the resting state network (RSN) in Figure 6B. Finally, Figure 6C displays the same results as brain map projections for the first metastable state (plotted in blue) in Figures 6A,B, because the second one is largely its complement.

Maximal metastability occurred bilaterally at lingual, calcarine, precuneus, occipital and cingulate cortices. The projection over the functional networks revealed maximum participation of the auditory and medial visual networks.

**Figure 7:**
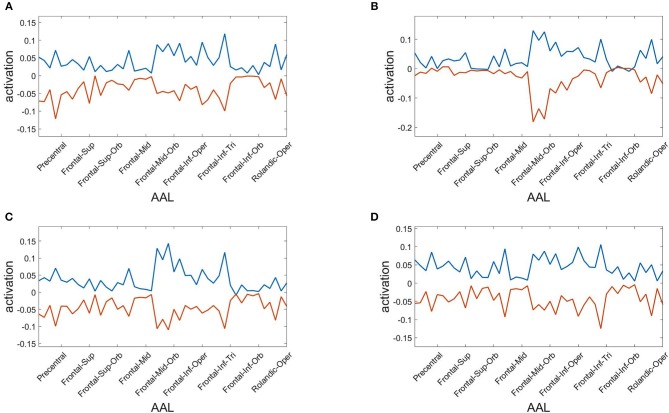
Anatomical AAL projections of the two metastable states obtained from optimizing RSA over 40 modules. Subjects: **(A)** #1, **(B)** #7, **(C)** #18, **(D)** #30.

**Figure 8:**
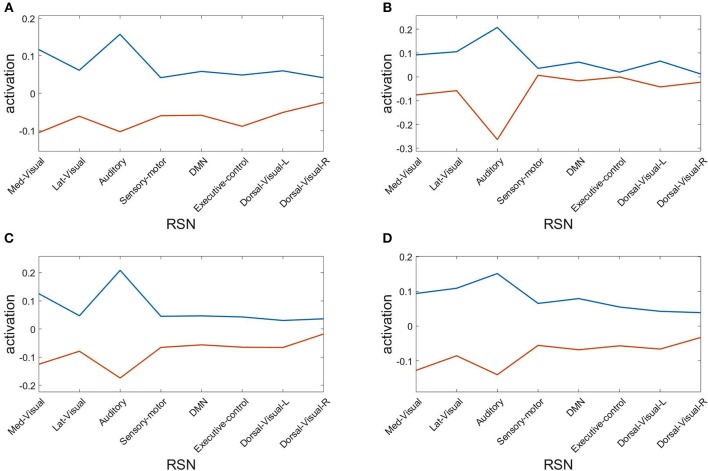
Resting state network (RSN) projections of the two metastable states obtained from optimizing RSA over 40 modules. Subjects: **(A)** #1, **(B)** #7, **(C)** #18, **(D)** #30.

Finally, we assess the robustness of the population analysis by breaking it down to the four selected subjects shown in Figures 3, 4. Figure 7 displays the AAL projections, whereas Figure 8 shows the corresponding RSN projections.

Both analyses indicate that these findings for single subjects are consistent with the population results.

## 4. Discussion

We introduced a novel framework to track the spatiotemporal dynamics of resting state fMRI BOLD signals. Specifically, a time dependent functional-structural brain mapping is achieved by combining the structural-functional brain hierarchical atlas (BHA) partitioning with the recurrence structure analysis (RSA). The combined method is applied to resting state fMRI BOLD signals and successfully identifies their corresponding time dependent metastable states, which are finally mapped to the anatomical brain structures and functional networks. Maximum metastability was found anatomically at lingual, calcarine, precuneus, occipital and cingulate cortices, which encompass both primary and high-order visual and auditory networks, together with the precuneus, that forming part of the default mode network is well-known to be one of the major functional hubs of the human brain. Although this is an observation rather than an interpretation, future studies are needed to really understand the brain organization of the metastable circuits at the large-scale.

The overall framework, in particular the time dependent aspect, relies fundamentally on Poincaré's recurrence theorem, which demands two conditions to be met for the underlying system under study. Specifically, if the system (typically an isolated or autonomous system) is volume preserving and has only bounded trajectories then for each open set (in phase space) there exist orbits that intersect the set infinitely often and are hence recurrent. It is critical to be cautious in drawing immediate interpretations of our results, however we will risk a tentative interpretation under the constraints made by the aforementioned theorem. To begin, we first assume that the brain (and associated processes) can be explained by some suitable complex multi-scale dynamical system. However, under the experimental fMRI condition in which the subjects are at rest, we will further assume that the underlying brain's dynamical system is autonomous (or at least approximately). Moreover, since fMRI BOLD relates to blood oxygenation and indirectly to local energy consumption through brain neural circuits, and thus correlates with neural activity, we assume that the values taken by the brain for its energy consumption are bounded. Finally, energy is not quantized, that is, it can take any value within this bounded domain. These assumptions satisfy the premises of the aforementioned theorem, which ultimately enables us to identifying time dependent recurrent states of resting state networks.

Under this setup, we find convergence toward an optimal segmentation of about 40 metastable states (for normalized data). This convergence may reflect preferred oxygen/energy levels (and switching between these levels) among all possible energy levels consumed across all structure-function brain modules. Thus at rest, it is likely that the energy is equally distributed globally across all networks (i.e., the whole brain), which in feedback entrains locally each module. However, each local module (or a set of few communicating modules) may have high transient use of energy consumption. These preferred energy levels and switching, indirectly reflect time dependent cognitive states mediated by the neural circuits of the resting state networks. To compare across all subjects we consider normalized data and following the Hausdorff clustering we find that across all subjects they share a common transition between two oxygen/energy levels (possibly interpreted as bistability). It is premature yet to draw any hard conclusions, however, we could extrapolate that there are two fundamental dwelling states that could represent a common homeostatic switching process that gateways the remaining energy levels, driving the dynamical transitions of the resting state structure-function network modules.

**Figure 9:**
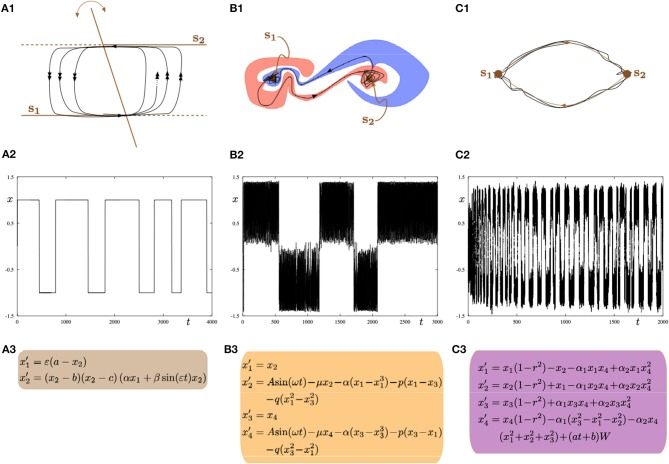
Three possible mathematical mechanisms to model switching dynamics: **(A1–A3)** slow-fast systems with a hysteresis loop; **(B1–B3)** systems with intermingled basins of attraction; **(C1–C3)** systems with a robust heteroclinic cycle. In all parts (left, central, right), the top panel shows a sketch of the phase space; the middle panel shows a time series of the minimal model representing one of the frameworks displaying alternating switches between two metastable states s_1_ and s_2_; the bottom panel shows minimal ODEs for a given framework. All equations are phenomenological but the resulting dynamics can be found in biophysical models of brain activity.

We subsequently contemplate on the dynamical systems point of view, the mechanistic alternatives that could explain switching between metastable states. Typically, within the brain mapping literature (specifically in computational modeling studies) it is often emphasized that transitions are explained via multistability. However, we would like to argue that again, it is crucial to be cautious since there are a multitude of mechanisms that can equally explain transition between states. For the sake of discussion, without wanting to exhaust all possible scenarios and avoiding modeling neurophysiological processes as it would go beyond the scope of the present manuscript, we showcase three canonical alternative mechanisms (as depicted in Figure 9). Moreover, we only focus on transitions between two states (to simplify the discussion). However, the proposed mechanisms can be easily extended to account for a larger number of metastable states. Nonetheless, these canonical mechanisms could be part of neural and hemodynamic biophysical models that explain, electrical-fMRI activity, such as those described via the dynamic causal modeling (DCM) framework (Friston et al., 2000), and/or other macroscopic modeling approaches (Breakspear et al., 2005; Chizhov et al., 2007; Rodrigues et al., 2007, 2009; Marten et al., 2009; beim Graben and Rodrigues, 2013).

The first canonical mechanism shown in Figure 9A is that of multiple-timescale dynamical systems, which are ubiquitous in neural modeling (Desroches et al., 2012, 2013, 2016). In this example, we consider a planar slow-fast system (Figure 9A3) with one slow variable *x*_1_ and one fast variable *x*_2_, where the separation of timescales between these variables is captured by the small timescale parameter 0 < ε≪1. The phase plane (*x*_1_, *x*_2_) (Figure 9A1) displays the fast nullcline or so-called *critical manifold* of the system, which consists of two horizontal lines and a third line intersecting the other two with a slowly-time-varying angle with respect to the vertical direction. This slowly-time-varying angle is arbitrary but could represent slow fluctuations inducing varying dwell times along a given attractor. The structure (i.e., fast nullcline) is the bifurcation diagram of the *fast subsystem* obtained by freezing the dynamics of the slow variable *x*_1_ (by setting ε = 0) and hence considering it as a parameter. In this context, the intersection points between the two horizontal components and the third component of the critical manifold correspond to transcritical bifurcation points (Rodrigues et al., 2016). Therefore, the fast subsystem possesses a hysteresis loop with two stable levels of activity, which in the full system (ε > 0 small) correspond to two metastable states **s_1_** and **s_2_** that a given trajectory (black curve) will visit recurrently in alternation. The single (respectively double) arrows along the trajectory correspond to slow (respectively fast) dynamics. Thus the transition between the slow and fast dynamics allows metastability. Parameter values for the simulation shown in Figure 9A2 are: *a* = 0.3, *b* = −1, *c* = 1, α = −2, β = −0.5, ε = 0.004.

A second canonical mechanism that captures switching dynamics between two metastable states is that of a dynamical system with two stable equilibria via so-called *intermingled or riddled basins of attraction* (Ding and Yang, 1996); this scenario is presented in Figure 9B. As illustrated in panel Figure 9B1 the basins of attraction of the both system's attractors can be arbitrary close to each other and they can even overlap in some region (and projections) of the phase plane of interest. This complex intermingling of basins of attraction enables the trajectory of the system to switch in a complex way between metastable states **s_1_** and **s_2_**, as shown in Figure 9B2. The specific model that we consider is formed by two coupled second-order differential equations with a sinusoidal forcing; see panel Figure 9B3. Parameter values for the simulation shown in panel (b2) are: *A* = 1.011, μ = 0.632, α = −4, *p* = 0.1, *q* = 0.005.

The third and last proposed mechanism shown in panels Figure 9C corresponds to a system possessing a robust *heteroclinic cycle* between two saddle equilibria (Rabinovich et al., 2008; Rodrigues and Labouriau, 2014; beim Graben and Hutt, 2015; Hutt and beim Graben, 2017). In this case, the metastable states are in fact saddles where the unstable manifold of each saddle connects to the other saddle, hence allowing for a stable robust heteroclinic cycle to exist. In this case, the system's trajectory as shown in Figure 9C2 displays recurrent switching between the two saddles that correspond to the two metastable states **s_1_** and **s_2_**. The heteroclinic cycle formed by these two states is stable and attracts trajectories. However, each new passage near one of the states brings the trajectory closer to one underlying saddle, hence passage times increase monotonically. However, fluctuations such as system noise (as is often the case in real systems) can disrupt or counteract these monotonic increase in passage times. The specific model is depicted in Figure 9C2, which is a four-dimensional dynamical systems on the 3-sphere ${𝕊}^{3}:=\left\{r^{2}=x_{1}^{2}+x_{2}^{2}+x_{3}^{2}+x_{4}^{2}=1\right\}$ possessing a robust stable heteroclinic cycle between two equilibria located at (0, 0, 0, ±1). The two saddles have only their last coordinate different. We add noise in the last equation with a time-dependent amplitude of the Brownian term and the resulting trajectory is depicted in Figure 9C2. Parameter values for the switching trajectory shown in panel (c2) are: α_1_ = 1, α_2_ = −0.1, *a* = 5·10^−5^, *b* = 0.33.

To conclude, there are potentially infinitely many models (and a multitude of dynamical mechanisms) that can equally explain the same fMRI BOLD observables. Thus a fundamental question for future research is what signatures within the data could potentially exclude cases and narrow down the possibilities enabling biophysical and parsimonious models to be derived. Finally, the present manuscript shows that is feasible to extract temporal information of the resting state networks, however it also opens up novel questions and avenues in brain mapping research.

## Data Availability

The datasets generated for this study are available on request to the corresponding author.

## Ethics Statement

The sample included 30 subjects from the MGH-USC Human Connectome Project. The research was performed in compliance with the Code of Ethics of the World Medical Association (Declaration of Helsinki). All subjects provided written informed consent, approved by the ethics committee in accordance with guidelines of HCP WU-Minn.

## Author Contributions

SR designed the project. PG did the recurrence analysis. AJ-M, ID, and JC did the data processing. MD did computational models. All authors wrote the paper.

### Conflict of Interest Statement

The authors declare that the research was conducted in the absence of any commercial or financial relationships that could be construed as a potential conflict of interest.

## Footnotes

^1^http://www.humanconnectome.org/

^2^Available for download at http://www.nitrc.org/projects/biocr_hcatlas/. A new Python version, that was developed during Brainhack Global 2017 – Bilbao, can be downloaded at https://github.com/compneurobilbao/bha

^3^A toolbox is freely available at https://www.researchgate.net/publication/317597921_RSA_Recurrence_Structure_Analysis_Toolbox_in_MATLAB
